# Protective Effects against the Development of Alzheimer’s Disease in an Animal Model through Active Immunization with Methionine-Sulfoxide Rich Protein Antigen

**DOI:** 10.3390/antiox11040775

**Published:** 2022-04-13

**Authors:** Adam S. Smith, Kyle R. Gossman, Benjamin Dykstra, Fei Philip Gao, Jackob Moskovitz

**Affiliations:** 1 Department of Pharmacology and Toxicology, School of Pharmacy, University of Kansas, Lawrence, KS 66045, USA; adamsmith@ku.edu (A.S.S.); gossman.kyle@ku.edu (K.R.G.); benjamindykstra@ku.edu (B.D.); 2 Protein Production Group, Shankel Structural Biology Center, University of Kansas, Lawrence, KS 66045, USA; gao@ku.edu

**Keywords:** methionine oxidation, active immunization, Alzheimer’s disease, oxidative stress

## Abstract

The brain during Alzheimer’s disease (AD) is under severe oxidative attack by reactive oxygen species that may lead to methionine oxidation. Oxidation of the sole methionine (Met35) of beta-amyloid (A*β*), and possibly methionine residues of other extracellular proteins, may be one of the earliest events contributing to the toxicity of A*β* and other proteins in vivo. In the current study, we immunized transgenic AD (APP/PS1) mice at 4 months of age with a recombinant methionine sulfoxide (MetO)-rich protein from *Zea mays* (antigen). This treatment induced the production of anti-MetO antibody in blood-plasma that exhibits a significant titer up to at least 10 months of age. Compared to the control mice, the antigen-injected mice exhibited the following significant phenotypes at 10 months of age: better short and long memory capabilities; reduced A*β* levels in both blood-plasma and brain; reduced A*β* burden and MetO accumulations in astrocytes in hippocampal and cortical regions; reduced levels of activated microglia; and elevated antioxidant capabilities (through enhanced nuclear localization of the transcription factor Nrf2) in the same brain regions. These data collected in a preclinical AD model are likely translational, showing that active immunization could give a possibility of delaying or preventing AD onset. This study represents a first step toward the complex way of starting clinical trials in humans and conducting the further confirmations that are needed to go in this direction.

## 1. Introduction

**Figure 1:**
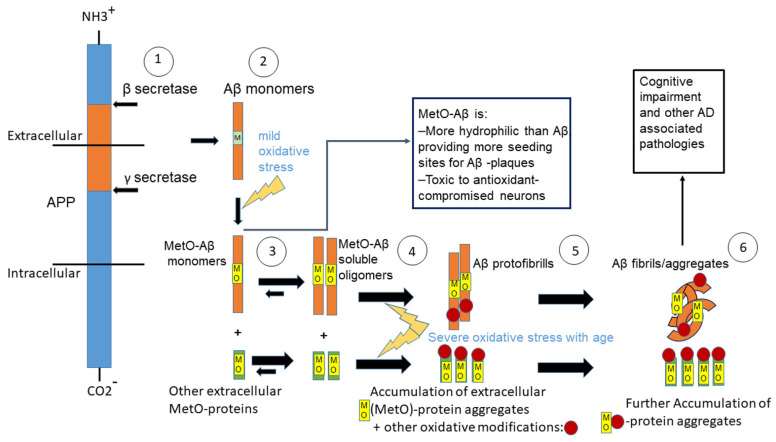
A graphical summary of the proposed events leading to MetO-A*β* and MetO-protein mediated toxicity in Alzheimer’s disease brain.

Oxidative stress appears in various biological systems through the production of reactive oxygen species that cause oxidative damage when the cellular antioxidant capacity is limited [1]. Consequently, oxidative stress plays a major role in normal aging as well as age-associated diseases such as the development of Alzheimer’s disease (AD) and other neurodegenerative diseases [2,3,4]. AD is characterized by an increase of lipid peroxidation and nucleic and protein oxidation levels [5]. Furthermore, pathological features that are associated with AD contain extracellular amyloid plaques, fibrillar A*β*, and neurofibrillary tangles [6]. Inflammation, activated microglia, and astrocytes are all involved in reactions in response to oxidative stress [6]. Amyloid plaques are considered to play a pivotal role in worsening AD [7]; however, soluble A*β* oligomers form before plaques and are considered the most neurotoxic and pathogenic form of A*β* [8,9]. Reactive oxygen species (ROS) can be produced by brain mitochondria, especially under stress and advance age, causing protein oxidation and cellular toxicity [10]. The importance of oxidative stress to the development and progression of AD is manifested by proposals for developing antioxidant-based therapies to alleviate AD-related pathologies [11,12]. A*β* contains a single methionine at position 35 of the protein sequence, located within a hydrophobic region. Thus, its polarity increases upon methionine oxidation [13]. Upon conditions of oxidative stress, the moiety of MetO-A*β* has been found to consist of up to 50% of the total A*β* of the plaques that are present in the AD brain [14,15,16,17]. It is hypothesized that at a younger age MetO-A*β* toxic effect is countered by a better immune system and through the upregulation of methionine sulfoxide reductase type A (MsrA), providing enhanced cellular antioxidant defense [13,18,19,20]. However, as the oxidative stress with advanced age is increased, while the activity of the MsrA and other antioxidants are decreased [21], MetO-A*β* and other, yet-to-be-determined MetO-containing proteins are predicted to gain toxicity towards neurons. Another important contributing factor to the enhanced toxicity of MetO-A*β* with age is its higher solubility in aqueous solution [13], providing better mobility and access of the MetO-A*β* to neurons throughout the hippocampal and cortical regions of the brain. Accordingly, clearance of A*β*-MetO from brain and other possible extracellular MetO-containing proteins may prevent or reduce AD-associated cognitive decline (see a summary figure, Figure 1). 

The membranal protein amyloid precursor protein (APP) is being cleaved by *β* and *γ* secretases (1), resulting in the release of A*β* monomers (2). Upon mild oxidative stress, methionine sulfoxide (MetO)-A*β* and other, yet-to-be-determined extracellular MetO-proteins are being accumulated (3). With time, these oxidized proteins become A*β* soluble oligomers and aggregated MetO-proteins (4). Upon severe age-dependent oxidative stress, the A*β* soluble oligomers are shifted towards protofibrils, and further aggregation of the MetO-proteins occurs. The proteins exhibit other oxidative modifications besides MetO (5). Finally, the A*β* fibrils/aggregates and the other oxidized proteins prompt the process leading to cognitive decline and other AD-associated pathologies. 

Immunization with A*β*-derived antigens or passive immunization with anti-A*β* antibodies has been shown to reduce A*β* burden in patients with AD and animal models of neurodegeneration. Although passive immunization using A*β* antibodies has been recently approved by the FDA in 2021 (Aduhelm TM, Biogen, Cambridge, MA, USA), the efficacy of this treatment is still debatable due in part to the relative mild positive effect of the treatment in preventing/inhibiting AD, while still raising concerns about possible adverse effects (e.g., encephalitis and rejection of the injected antibodies by the immune system over time). Thus, an alternate possibility is to use replacement antigens based on non-A*β* proteins that might cause the clearance of oxidized toxic proteins (i.e., MetO-A*β* and other extracellular MetO-proteins) without causing the previously observed adverse effects, while possessing stronger effectiveness. Accordingly, active immunization using an oxidized *Zea mays* Met-rich protein is suggested to resolve all these concerns while alleviating the phenotypes of AD in mice. This approach clears only an aberrant form of A*β*: the one containing MetO and possibly other extracellular MetO-proteins that contribute to the pathogenesis of AD. 

The hypothesis of the current research is that, compared with the controls, the active immunization of APP/PS1 mice with MetO-rich protein will inhibit short-and long-term memory along with molecular phenotypes that are associated with AD. For this purpose, the levels of the following parameters are determined: the levels of total A*β*_42_ (both in blood-plasma and whole brain); the levels of amyloid plaque burden in the hippocampal and cortical regions of the brain; the levels of activated microglia (as determined by Iba1 expression), and the levels of MetO-protein in astrocytes. Finally, the cellular distribution and localization of the important antioxidant regulator Nrf2 [22] is determined. The overall impact of the presented data on the possibility of using MetO-rich antigen immunization for AD treatment in humans is discussed.

## 2. Material and Methods

### 2.1. Antibodies

Anti-A*β*_42_ antibody was purchased from BioLegend (San Diego, CA; USA, 6E10 monoclonal antibody for the detection of amyloid plaque burden in brain sections of APP/PS1 mice at 10 months of age, when most, if not all, of the human APP is cleaved into A*β*). Anti-Iba1 and anti-Nrf2 antibodies were purchased from GeneTex (Irvine, CA, USA). Anti-mouse/rabbit IgG HRP-conjugated antibody was purchased from Santa-Cruz Biotechnology (Santa Cruz, CA, USA). Anti-rabbit/mouse fluorescent-labeled antibody was purchased from Thermo Fisher Scientific (Wathham, MA, USA). The original rabbit anti-MetO antibody was produced in our lab as previously described [23]. 

### 2.2. MetO-Rich Protein (Antigen) Production

**Figure 2:**
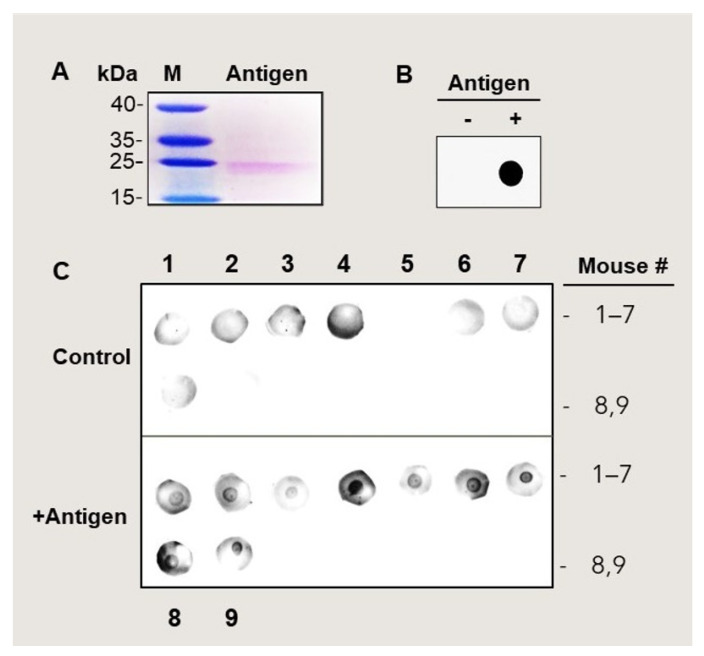
Antigen-injected mice produce antibody against MetO-rich protein in blood-plasma. (**A**). The MetO-rich protein (Antigen) was produced and purified according to the procedures described under the “Materials and Methods” section. The purified antigen was separated on SDS-gel-electrophoresis that was stained with Coomassie brilliant blue (the limited protein staining level is due to the relatively low presence of basic amino acids in the Met-rich protein sequence). M, molecular mass markers; kDa, molecular mass numbers. (**B**). A dot-blot analysis using the MetO-rich protein as the loaded protein (Antigen) and rabbit anti-MetO antibody (1:1000 dilution) as the primary antibody. (**C**). A dot-blot analysis using the MetO-rich protein, as the loaded protein (Antigen), probed with plasma moiety (used as the primary antibody, 1:100 dilution, 1 h incubation time) from 10-month-old mice. Only the antigen-injected mice showed positive reactions with the antigen (a round black reaction in the middle of the blot), while the controls were all negative. Numbers 5 and 9 of the control blots had almost no background reactions.

The recombinant MetO-rich protein was produced in a similar way to the previous 6His-tagged MetO-rich protein [23], except for the use of Maltose Binding Protein (MBP) as the fusion protein instead of the 6His-tag adduct. This change dramatically enhanced the production and purification yield of the recombinant protein. Codon-optimized DNA of the *Zea mays* Met-rich protein (DZS18 protein) was synthesized from Integrated DNA technologies (Coralville, IA, USA) and was subcloned into the pTBMalE plasmid [24]. The resulting 6His-MBP-DZS18 construct was confirmed by DNA Sanger sequencing. Competent bacterial cells (BL21(DE3)) pRARE were transformed with the 6His-MBP-DZS18 construct, and positive clones were selected on agar plate supplement with 100 µg/mL ampicillin and 34 ug/mL of chloramphenicol. Protein was induced at log phase in LB media + antibiotic with 0.4 mM IPTG for 3 h at 37 °C. Thereafter, the cells were pelleted by centrifugation and frozen at −80 °C. After the freeze/thaw cycle, the cells were disrupted by sonication on ice. Following high-speed centrifugation (10,000× *g*) the supernatant was collected and the protein was purified through Ni affinity chromatography. Briefly, the overexpressed 6His-MBP-DZS18 protein (596 aa, 65,833 DA) was purified to homogeneity from the supernatant by affinity purification using Ni^2+^-NTA resin (Qiagen, Germantown, MD, USA), according to the manufacturer’s protocol. The final buffer composition of the purified 6His-MBP-DZS18 protein was: 50 mM Tris-HCl, 0.5 M NaCl, and 500 mM imidazole. Then, the protein was digested with trypsin (trypsin: 6His-MBP-DZS18 = 1:10 (*w/w*) overnight at 37 °C. The major resulting protein had 202 AA with a 22,629 Da mass and the minor one (following one more cleavage) had 163 AA with an 18,189 Da mass. The digested protein material was purified on SEC75 with a mobile phase buffer containing PBS + 1 M guanidine hydrochloride, and the resulting peak protein was the major protein that was purified to homogeneity. Then, the protein was concentrated with a 10,000 DA cut-off centricon to remove any small remaining peptides. The final concentration of the protein was 1 mg/mL, and its purity was confirmed by SDS-gel-electrophoresis followed by Coomassie brilliant blue (Thermo-Fisher Scientific, Waltham, MA, USA) staining (Figure 2A; the limited protein staining level is due to the relative low presence of basic amino acids in the Met-rich protein sequence). Thereafter, the concentrated protein was then oxidized overnight at room temperature by adding to the protein mixture 200 mM of H_2_O_2_. The H_2_O_2_ decomposes with time and the protein was kept at −80 °C until use. Following MALDI-TOF analysis, the molecular mass of the purified oxidized protein was a 23,330 Da mass (predicted 202 AA with a 22,629 Da mass), indicating that 44 out of the protein’s 48 Met residues were oxidized. To validate the immunogenicity of the MetO-rich protein, the protein was injected into a rabbit to create poly-clonal anti-MetO antibody as previously described [23]. Indeed, the resulting antibody showed specificity towards Met-oxidized proteins (Figure 2B), similarly to the previously described production of the anti-MetO antibody, using 6His-tagged MetO rich protein as the antigen [23]. 

### 2.3. Mice Studies and Immunization Procedure 

A commonly used AD model mice (APP/PS1 (APPswe, PSEN1de9) 85Dbo, 18 female mice at age of ~1–2 months) were purchased from Jackson’s lab. All animals were housed in the animal care facility of the University of Kansas under pathogen-free conditions. No more than five animals were present in one cage, where they were housed under a 12-h light and dark cycle with free access to food and water. Following the completion of the immunization procedures and their reaching the age of 10 months, the animals were euthanized in accordance with the American National Institute of Health’s animal care and use-approved procedures and in agreement with the University of Kansas’ animal care unit-approved protocol (# 144-01). The mice were immunized with the MetO-rich protein (antigen). The mice were divided into two groups, in which one was injected with the vehicle only, i.e., Alum adjuvant (Sigma-Aldrich, St. Louis, MO, USA) (control group), and one with the vehicle and antigen (experimental group). Specifically, at ~3–4 months of age, the control group (nine females) was injected with Alum adjuvant only, while the experimental group (nine females) was injected with Alum plus the antigen (50 μg/mouse). Accordingly, two additional booster injections were performed in the following month (2-week intervals). This immunization model is a common practice in rodent vaccination. Thus, the whole immunization was completed at 4–5 months of age for all animals. Then, at 10 months of age the bio-behavioral testing was performed, followed by the postmortem analyses. Accordingly, the details for the experimental paradigm that shows the timetable of the experiments are depicted in Table 1. 

### 2.4. Mice Bio-Behavior Analyses

APP/PS1 mice show deficiencies in their memory and learning capabilities starting at 6 months of age. Thus, allowing time for the active immunotherapy to be effective. The data acquired from the bio-behavioral analyses was tested for significance using one-way analysis of variance (ANOVA) with post hoc Tukey’s test. 

### 2.5. Y-Maze 

The Y-maze test is a simple and common maze used to assess spatial working memory in mice by measuring their willingness to explore new environments [25]. Mice were placed into the center of the 3-armed Y-maze with one arm obstructed and they were allowed to freely explore the two non-obstructed arms for 5 min. This served as a familiarizing training trial. Thirty-minutes later, mice were reintroduced into the Y-maze for a 5-min period, and in this trial the full arena was accessible. Total arm entries, as well as time spent in the novel and familiar arms, were recorded. An extended period in the novel arm beyond chance is indicative of sustained cognition and short-term spatial memory, as the animals must remember which arm was not accessible during the training trial, and thus this was a novel environment to explore.

### 2.6. Morris Water Maze (MWM)

MWM is one of the most common behavioral tests used to determine hippocampal spatial short- and long-term memory deficits, particularly those observed in AD mouse models. MWM test was performed a week after the completion of the YMSA test [25]. The training paradigm for the hidden platform version of the MWM consisted of four trials each day (at an interval of 15 min) for 5 consecutive days. The capacity of mice to retrieve and retain the learned location of the platform was assessed during a probe trial, which was carried out 24 h after the completion of training on the fifth day. 

### 2.7. Monitoring Total Aβ_42_ Levels in Mouse Blood-Plasma and Brain

At 10 months of age, the mice were perfused with PBS, samples of their blood were collected, and their brains were extracted. The soluble blood-plasma was separated and collected by centrifugation for 15 min at 2000× *g* in the presence of 1 mM EDTA. Soluble proteins from one hemisphere of each brain were obtained following guanidine-HCl extraction, as described by the ELISA kit protocol for A*β*_42_ (Thermo-Fisher Scientific; Catalog **#** KHB3441). The corresponding levels of A*β*_42_ in blood-plasma and the total brain of each mouse were measured according to the protocols provided by the same ELISA kit.

### 2.8. Immunohistochemistry and Staining of Mouse Brains’ Slices

The complementary hemisphere of each postmortem PBS-perfused brain was fixed in 4% paraformaldehyde in PBS at 4 °C and then flash-frozen by exposure to dry ice. Coronal sections (30 μm) were cut using a cryotome and transferred to gelatin-coated glass slides. For the detection of β-pleated sheet conformation of amyloid, sections were stained with Thioflavin-S dye solution [1%], rinsed in water, dehydrated through graded ethanol, cleared with xylene, and finally mounted with permount [26]. For immunohistochemistry staining, sections were blocked with 3% (*w/v*) gelatin in PBS (1 h at 37 °C), treated with 0.1% Triton X-100 in PBS (15 min at 23 °C), and reacted (overnight at 4 °C plus 1 h at 23 °C) with the selected primary antibody. After rinsing in PBS, the sections were incubated (2 h at room-temp) with either primary antibody-matched fluorescent dye-labeled secondary antibodies (Alexa 568 goat anti-rabbit or Alexa 488 goat anti-mouse; Thermo Fisher Scientific) or HRP-labeled secondary goat anti-mouse/rabbit antibody (Santa-Cruz Biotechnology). Then, the sections were rinsed with PBS followed by Tris-EDTA buffer, mounted, viewed, and analyzed by using a light microscope. Quantification of the emitted signal per brain section was achieved by calculating the mean signal coverage per same brain area using NIH Image-J program (Figures 4–6) or manually counting the nuclear Nrf2 signal per same-size area (Figure 7) in the three selected brain regions (HPC, RSC, and EC, *n* = 4 brain slices per tested mouse).

### 2.9. Quantification and Statistical Analyses

Measured data are presented as the mean ± standard errors. All statistical analyses were performed using Sigma Plot 14. Data were analyzed with a two-tailed Student’s *t*-test and one-way ANOVA followed by Tukey’s multiple-comparisons test. *p* values equal to or lower than 0.05 were considered to indicate a statistically significant difference. 

## 3. Results

### 3.1. Production of Anti-MetO Antibody in Rabbit and Mice

The newly synthesized purified antigen (Figure 2A) was injected into a rabbit to reproduce and validate its ability to create anti-MetO antibody, as previously described [23]. Indeed, only antigen-immunized rabbit showed a strong reaction against the antigen, following a dot-blot analysis (Figure 2B) and western blot analysis (Figure S1). The dot-blot analysis is the preferred method for monitoring the anti-serum against MetO since the antigen structure is closely mimicking its injected form (in an SDS-gel electrophoresis-western blot analysis the antigen is further modified by SDS). At 4 months of age, the APP/PS1 mice were injected with either the adjuvant only (control) or with the mixture of the adjuvant and antigen (experimental). An additional two boosts of injection were given in the following month in 2-week intervals. At 10 months of age, all the antigen-injected mice showed positive reactions against the antigen, while all the mice of the control group exhibited negative reactions (Figure 2C). These results confirm that all the antigen-injected mice were successfully immunized against the antigen and obtained significant levels of anti-MetO antibody in sera throughout their life up to their euthanasia at 10 months of age. 

### 3.2. Bio-Behavioral Data

#### 3.2.1. Y-Maze

**Figure 3:**
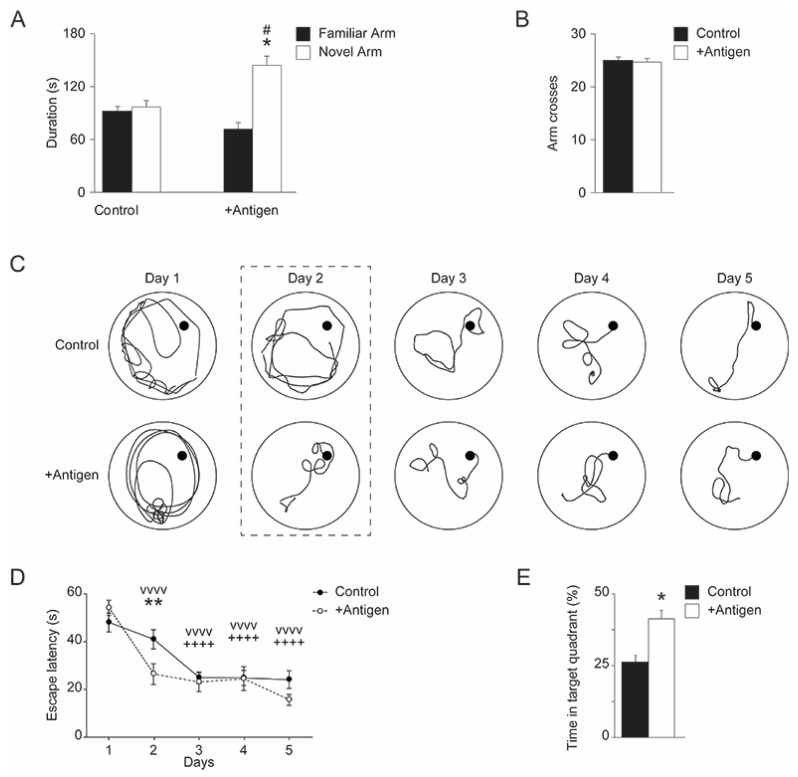
Vaccinated APP/PS1 mice have improved short- and long-term spatial memory. (**A**) Antigen-immunized APP/PS1 mice have a novel arm preference in a forced Y-maze test but control APP/PS1 do not, displaying improved short-term memory after vaccination. The details of the experimental procedures are listed under “Materials and Methods”. (**B**) Arm crossing is not affected by vaccination, indicating age-typical locomotion. (**C**) Morris Water Maze (MWM) analyses (the details of the experimental procedures are listed under the Section 2). These are representative schematics of swimming patterns performed by mice over the 5-day training period of the MWM. These swimming patterns illustrate that antigen-immunized APP/PS1 mice improve their escape latency after a single day of training, while control mice require repeated training days to improve in this task (as reflected in Day 2). (**D**) This is a statistical presentation of the significant effect on the antigen-immunized mice’s reduced escape latency during the second day of training, while the control mice do not improve in this task until day 3. This demonstrates an improved learning for this long-term spatial memory task in antigen-immunized mice (**E**) Antigen-immunized mice also spend more time swimming in the target quadrant (after the platform is removed from the pool) compared to controls, who are only performing at chance. Standard deviation is shown for each group of mice. Data were acquired from all tested mice (*n* = 9 per each group of mice). Statistical significances were assessed for the acquired data using one-way analysis of variance (ANOVA) with post hoc Tukey’s test: # *p* < 0.05 familiar vs. novel arm within condition; * *p* < 0.05, ** *p* < 0.01 vs. control; ^vvvv^ *p* < 0.0001 vs. day 1 training for antigen-immunized mice; ^++++^ *p* < 0.0001 vs. day 1 training for control mice.

Cognitive impairment has been validated for APP/PS1 mice as early as 9 months of age. This includes short- and long-term spatial memory deficiencies. Thus, we subjected the control and antigen-immunized mice to both Y-maze and Morris water maze tests at 10 months of age. In a Y-Maze task, the controls averaged about 103 s in the novel arm, which is right at the level of chance for a 300 s or 5 min test. Antigen-immunized mice spent significantly more time exploring a novel arm compared to the controls (One-way ANOVA, F_(1,16)_ = 9.022, *p* = 0.008; Figure 3A). Total arm crosses did not appear to be statistically different between groups (Figure 3B), so there was no effect of treatment on locomotor behavior. Thus, antigen-immunized mice had improved short-term spatial memory and a novel arm preference, while the control mice failed to differentiate between familiar and novel arms.

#### 3.2.2. Morris Water Maze (MWM)

Over a 5-day training period, all animals eventually reduced their escape latency time (One-way ANOVA, F_(4,64)_ = 29.073, *p* < 0.001; Figure 3C,D). However, antigen-immunized mice improved their escape latency faster than the controls, dropping time significantly by the second day of training, while it required repeated days of training for the controls to reduce escape time (One-way ANOVA, F_(4,64)_ = 2.942, *p* = 0.027). Furthermore, during the probe day (when the platform was removed from the MWM), the control mice spent 26.7% of the total time swimming in the target quadrant, indicating a failure to form long-term memory of the platform location (as 25% was the predicted chance percentage of time swimming in any given quadrant) (Figure 3E and Video S1). This failure in long-term spatial memory replicates published work in APP/PS1 mice at this age [27]. Interestingly, the antigen-immunized females spent significantly more time in the target quadrant than the controls (two-tailed *t*-test *t*_(16)_ = 2.615, *p* = 0.019; Figure 3E and Video S2). Together with the Y-maze data, it appears the antigen vaccination significantly slowed the age-dependent cognitive decline observed in short-term and long-term memory, typically demonstrated in APP/PS1 mice.

### 3.3. Aβ Levels in Blood-Plasma and Brain

**Figure 4:**
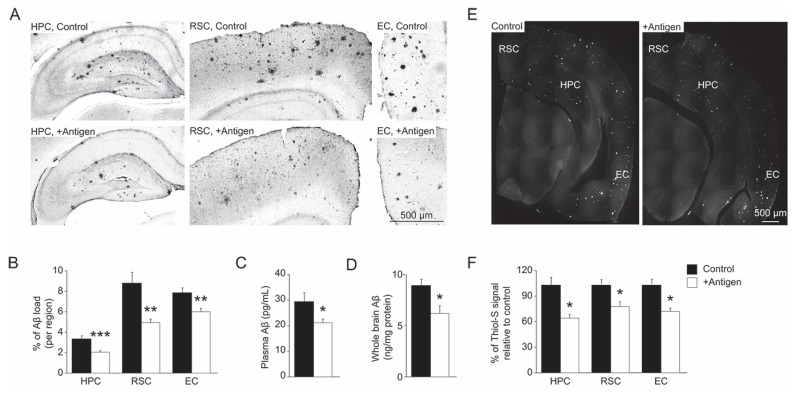
Reduction of A*β*_42_ in blood-plasma and brain, and reduction of amyloid plaque burden in brains of antigen-injected mice. (**A**). Hemisphere brains of post-mortem mice (control and antigen-immunized) were dissected, and immunohistochemistry analyses were performed to detect A*β*_42_, according to the procedure described under the Section 2. The data shows a decline in the levels of A*β*_42_ in the three brain regions (HPC, hippocampus; RSC, retrosplenial cortex, and EC, entorhinal cortex) of the antigen-immunized as compared with the control mice. (**B**). Quantification of the data presented in Panel A using NIH-Image J program. Quantification of A*β*_42_ in blood-plasma (**C**) and post-mortem brain extracts (**D**) of antigen-injected and control mice using ELISA kit according to the procedure described under the Section 2. (**E**). Thioflavin-S staining of the post-mortem mouse brains according to the procedure described under the “Materials and Methods” section. Lower plaque-burden was observed in the antigen-injected versus control mice. (**F**). Quantification of the data depicted in panel E using NIH Image-J program. In all of the graphs, black and white bars represent control and antigen-injected mice, respectively. Standard deviation is shown for each averaged bar. Data were acquired from all tested mice for panels C and D (*n* = 9 per each group of mice) and five mice per each tested group of mice for panels B and F. Statistical analyses were performed using two-tailed student *t*-test with the following *p* values: *, *p* < 0.05; **, *p* < 0.01, and ***, *p* < 0.001.

At 10 months of age, the levels of A*β_42_* in blood-plasma and whole-brain extracts of postmortem mice were analyzed. Compared to the control group, the antigen-immunized mice showed an average of 31% and 28% decline in the level of blood-plasma and whole-brain A*β_42_*, respectively (Figure 4C,D, two-tailed *t*-test, *t*_(11)_ = 2.495 (plasma), *p* = 0.02; *t*_(11)_ = 2.313 (brain), *p* = 0.04). Immunohistochemistry analyses of the hippocampal and cortical regions of the brain (hippocampus (HPC); retrosplenial cortex (RSC) and entorhinal cortex (EC)) demonstrated similar results (Figure 4A,B). Specifically, compared to controls, the antigen-immunized mouse brain regions exhibited an average decline in the levels of A*β_42_* as follows: 38% in the HPC, 42% in the RSC, and 29% in the EC (Figure 4B, two-tailed *t*-test, *t*_(13)_ = 6.432, *p =* 0.0002 for HPC; two-tailed *t*-test, *t*_(12)_ = 3.883, *p =* 0.003 for RSC, and 3. 546, *p =* 0.002 for EC). These data were corroborated by Thioflavin-S staining that enables the detection of β-pleated sheet conformation of amyloid (although this staining is less specific relative to the immunohistochemistry analysis). Accordingly, compared to the controls, the antigen-immunized mouse brain regions exhibited an average decline in the levels of Thioflavin-S staining as follows: 34% in the HPC, 20% in the RSC, and 26% in the EC (Figure 4E,F, two-tailed *t*-test, *t*_(19)_ = 2.488, *p =* 0.04 for HPC; *t*_(19)_ = 2.107, *p =* 0.02 for RSC; *t*_(19)_ = 2.405, *p =* 0.02 for EC). Overall, these observations show that the levels of A*β* in both blood-plasma and brains of the antigen-injected mice were significantly reduced compared to the control mice.

### 3.4. Levels of MetO-Proteins in Brain Cells

**Figure 5:**
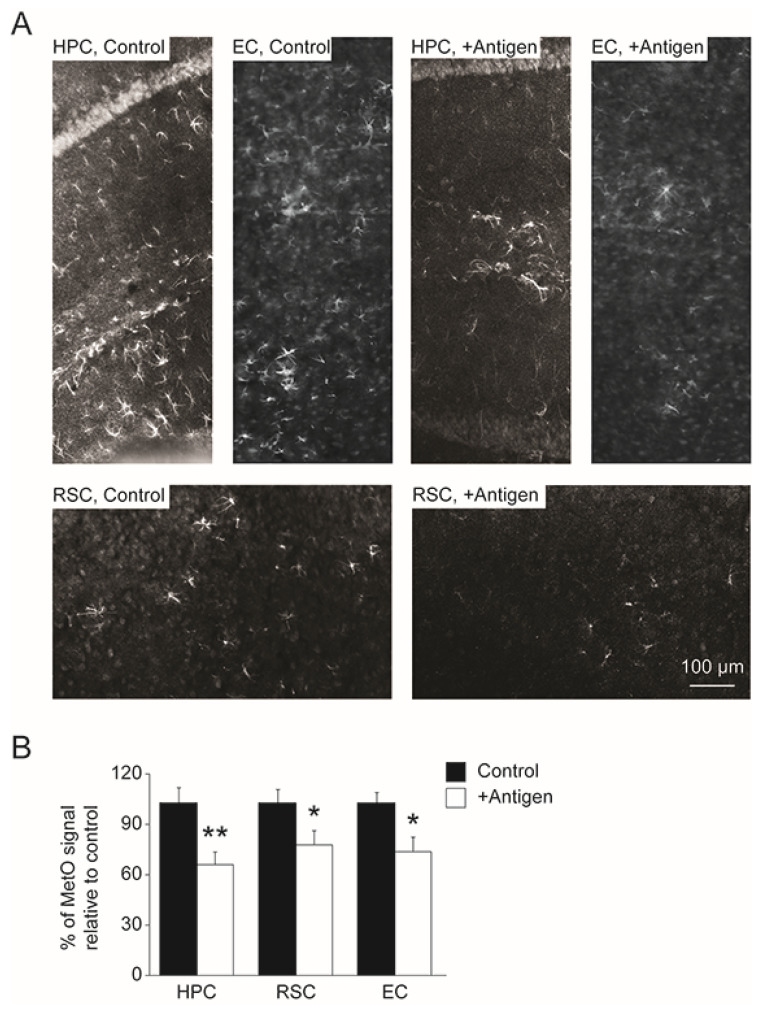
Reduction of MetO-protein levels in brain astrocytes in antigen-injected mice (**A**). Hemisphere brains of post-mortem mice (control and antigen-immunized) were dissected, and immunohistochemistry analyses were performed to detect MetO-proteins according to the procedure described under the Section 2. The data shows a decline in the levels of MetO-proteins in the three brain regions (HPC, hippocampus; RSC, retrosplenial cortex, and EC, entorhinal cortex) of the antigen-immunized compared with control mice. (**B**). Quantification of the data presented in Panel A using NIH-Image J program. Black and white bars represent control and antigen-injected mice, respectively. Standard deviation is shown for each averaged bar. Data were acquired for five mice per tested group of mice. Statistical analyses were performed using two-tailed student *t*-test with the following *p* values: *, *p* < 0.05, and **, *p* < 0.01.

Besides the direct effect of the vaccination on the levels of A*β* deposits in brain, there were other indirect consequences of the treatment at the cellular level. The cellular antioxidant capacity of brain cells was compromised upon enhanced oxidative stress, as has been demonstrated in AD [1]. For example, astrocytes are known for their high antioxidant capability in protecting neuronal cells from oxidative damage. Thus, a reduction in this function may be reflected by the accumulation of oxidized proteins (e.g., MetO-proteins) in these cells. Accordingly, vaccination-mediated alleviation of oxidative stress may be evident by the existence of lower levels of MetO-proteins in the brain. Indeed, as shown in Figure 5, the control mice had higher levels of MetO-proteins in brain astrocytes, compared to the antigen-injected mice. Specifically, compared to controls, the antigen-immunized mouse brain regions exhibited an average decline in the levels of MetO-proteins as follows: 36% in the HPC, 25% in the RSC, and 23% in the EC (Figure 5A,B, two-tailed *t*-test, *t*_(17)_ = 3.142, *p =* 0.006 for HPC); two-tailed *t*-test, *t*_(18)_ = 2.613, *p =* 0.04 for RSC; *t*-test, *t*_(18)_ = 2.192, *p =* 0.02 for EC). 

### 3.5. Indices of Gene Regulation in Brain Cells

**Figure 6:**
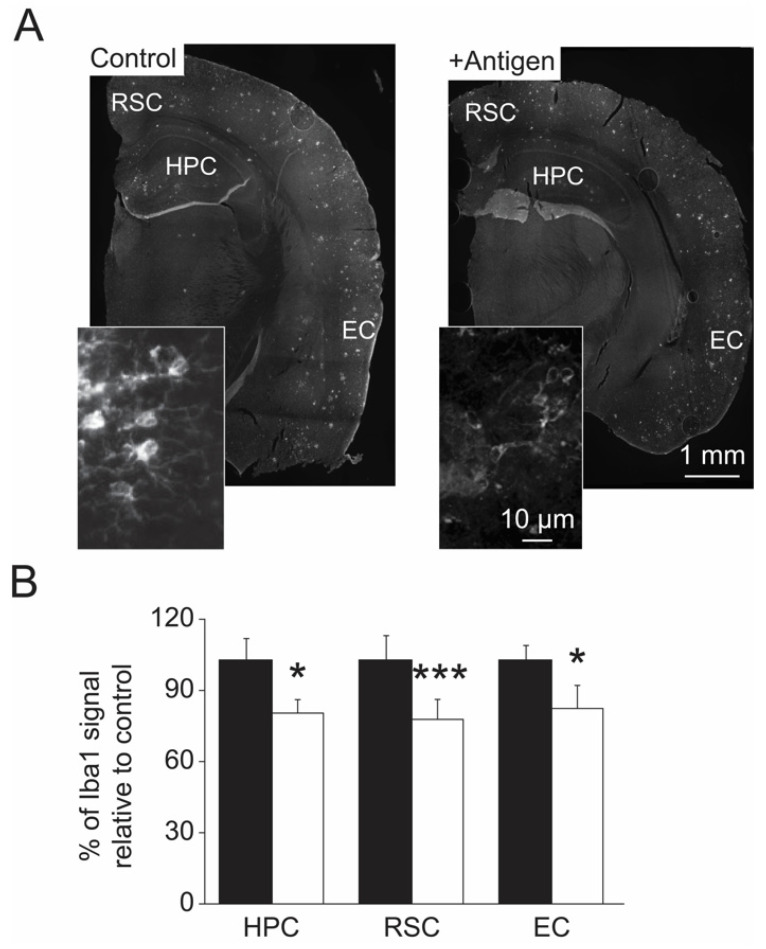
Reduced activation of microglia in antigen-injected mice. (**A**). Hemisphere brains of post-mortem mice (control and antigen-immunized) were dissected, and immunohistochemistry analyses were performed to detect Iba1 according to the procedure described under the Section 2. The data show a decline in the levels of Iba1 of neuronal cells in the three brain regions (HPC, hippocampus; RSC, retrosplenial cortex, and EC, entorhinal cortex) of the antigen-immunized compared with control mice. (**B**). Quantification of the data presented in Panel A using NIH-Image J program. Black and white bars represent control and antigen-injected mice, respectively. Standard deviation is shown for each averaged bar. Data were acquired from five mice per tested group of mice. Statistical analyses were performed using two-tailed student *t*-test with the following *p* values: *, *p* < 0.05; ***, *p* < 0.001.

Activation of microglia assist in the clearance of A*β* deposits in the brain and thus their activation level corelates with A*β* level. Therefore, a reduction of the level of activated microglia upon antigen vaccination is expected since lower A*β* deposits were observed in the antigen-vaccinated mice (Figure 4). Accordingly, compared to the control mice, all of the tested brain regions of the antigen-vaccinated mice showed lower levels of activated microglia (as judged by the levels of the activated microglia marker protein Iba1) (Figure 6A,B). Specifically, compared to the controls, the antigen-immunized mouse brain regions exhibited an average decline in the levels of Iba1-protein as follows: 25% in the HPC, 24% in the RSC, and 26% in the EC (Figure 6A,B, two-tailed *t*-test, *t*_(12)_ = 3.077, *p =* 0.01 for HPC; *t*-test, *t*_(15)_ = 7.338, *p =* 0.0001 for RSC; *t*-test, *t*_(12)_ = 2.743, *p =* 0.02 for EC). 

**Figure 7:**
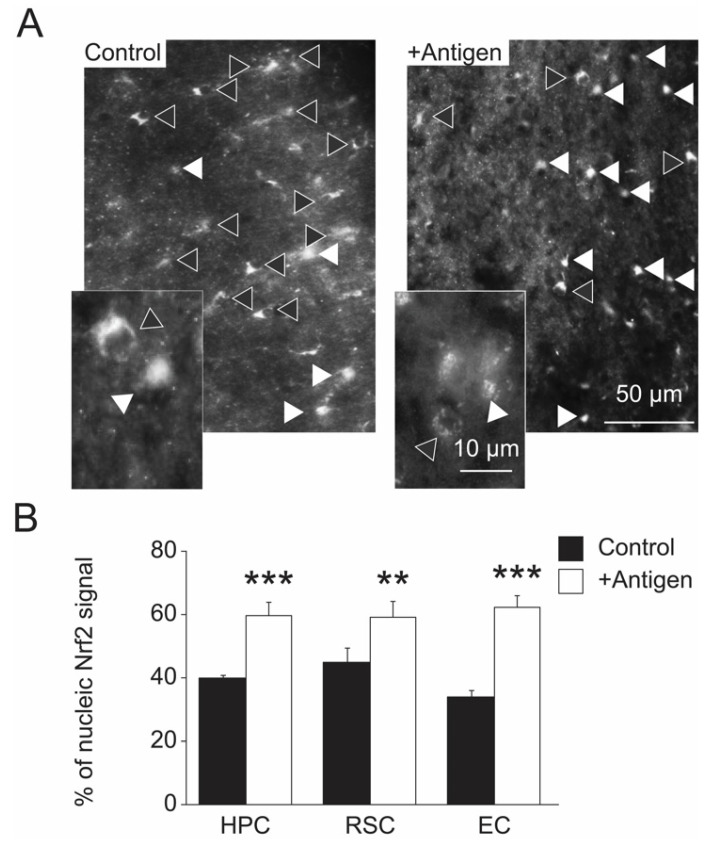
Increased nuclear localization of Nrf2 in antigen-injected mice. (**A**). Hemisphere brains of post-mortem mice (control and antigen-immunized) were dissected, and immunohistochemistry analyses were performed to detect Nrf2 according to the procedure described under the Section 2. The data shows an increased nuclear localization within astrocytes of Nrf2 in a representative image of the EC region of the antigen-immunized compared to the control mice. A similar pattern of Nrf2 localization ratio between the two mouse groups was observed for the HPC and RSC regions (images are not shown). The white triangle symbols point to cells harboring nuclear Nrf2 and the black-filled triangle symbols point to cells harboring cytosolic Nrf2 (**B**). Quantification of the data presented in Panel (**A**) by manually counting the nuclear Nrf2 signal per same-size area in the three selected brain regions. Black and white bars represent control and antigen-injected mice, respectively. Standard deviation is shown for each averaged bar. Data were acquired from five mice per tested group of mice. Statistical analyses were performed using two-tailed student *t*-test with the following *p* values: ***, *p* < 0.001 and **, *p* < 0.01.

An important transcription factor that regulates the expression of several antioxidant genes is Nrf2 [22]. This function of Nrf2 is mediated through its translocation to the nucleus from the cytoplasm, causing an enhanced transcription of several gens, including antioxidant genes [22]. Hence, a relatively high nucleic localization of Nrf2 is beneficial in providing a strong cellular antioxidant protection. Interestingly, compared to the control mice, the antigen-immunized mice demonstrated a higher rate of nuclear localization of Nrf2 mainly in astrocytes (Figure 7A,B), suggesting a positive effect of the antigen on Nrf2 function through a yet-to-be-discovered mechanism. Specifically, compared to the controls, the antigen-immunized mouse brain regions exhibited an average rate increase of Nrf2-nuclear localization as follows: 59% vs. 39% in the HPC, 57% vs. 44% in the RSC, and 60% vs. 31% in the EC (two-tailed *t*-test, *t*_(13)_ = 7.882, *p =* 0.0004 for HPC; *t*-test two-tailed *t*-test, *t*_(17)_ = 3.135, *p =* 0.006 for RSC; *t*-test, *t*_(13)_ = 7.619, *p =* 0.0005 for EC). These data correlate with the observation of lower MetO-protein levels in astrocytes of the antigen-immunized mice (Figure 4). 

## 4. Discussion

Accumulation of extracellular MetO-proteins as a biomarker for the presence of AD has been described in both postmortem human brains [16] and, recently, in live human blood-plasma [28]. Thus, it was suggested that these MetO-proteins, including MetO-A*β,* are involved in the etiology of AD. To provide evidence for this hypothesis transgenic AD model (APP/PS1), mice were immunized with MetO-rich protein antigen and evaluated for their cognitive function and the expression of molecular biomarkers that are associated with AD. Vaccination of the APP/PS1 mice with the MetO-rich protein antigen (Figure 2A) rendered a relatively long-lasting production of anti-MetO antibody in their blood-plasma (Figure 2C). In addition, rabbit immunization with this antigen resulted in the production of anti-MetO antibody as well (Figure 2B). Thus, the ability to induce the creation of a long-lasting specific anti-MetO antibody in two different mammalian species (mouse and rabbit) without obvious signs of adverse effects supports the foundation for the potential use of this vaccination in humans. Following this active immunization of the APP/PS1 mice and compared to the control group, the antigen-injected mice showed significantly better cognitive functions, as judged by the bio-behavioral analyses performed at 10 months of age (Figure 3). The APP/PS1 mice start to show decline in cognitive function around 7 months of age [29,30]. Therefore, the demonstrated positive effect of the vaccination supports the hypothesis that the clearance of extracellular MetO-proteins (including MetO-A*β*) from the brain alleviates pathologies that are affecting learning and memory deficits that are typical characteristics of AD. It is yet to be determined what are the mechanisms by which the immune system removes MetO-proteins from brain. There are several known and yet-to-be-discovered possibilities that could explain the clearance of MetO-proteins by the current active immunization. For example, since only 0.1% of the circulating antibodies can penetrate the brain and overcome the blood-brain barrier (BBB), it was for a long time speculated that the immune system has a way of protecting the brain not just by activating glia cells but also through the humoral immunity. Indeed, recently, CD4^+^ T-helper cells have been shown to mediate the opening of the BBB upon stimulation with any antigen. Specifically, following the presentation of an antigen to CD4^+^ T- helper cells, they are recruited to the central nervous system (CNS) and lead to BBB aperture, thus allowing antibody passage [31]. Accordingly, active immunization with the MetO-antigen may recruit CD4+ T-helper cells to the CNS, leading to the subsequently produced anti-MetO antibody gaining access to the brain through the CD4+ T-helper cells-mediated opening of the BBB. 

Another possible explanation for the observed beneficial effect of the current immunization on alleviating phenotypes of AD is the ability of the systemic circulating anti-MetO antibodies to quench the levels of MetO-A*β* in fluids outside the brain (e.g., cerebrospinal fluid (CSF) and blood plasma). Under this situation, the brain MetO-A*β* proteins (and perhaps non-oxidized A*β* proteins as well) will be excreted from the CNS towards the systemic fluids to balance the lower levels of A*β* in the CSF and plasma. Hence, a consistent systemic immuno-clearance of MetO-A*β* is expected to drain the levels of A*β* in the brain, leading to lesser expression of AD markers. 

The data presented in Figure 3 strongly suggest that, compared to the controls, the antigen-immunized mice exhibit impressively better short- and long-term memory (as judged by the Y-maze and MWM analyses), while demonstrating superior learning capabilities (as judged by the MWM analyses). To the best of our knowledge, the current non-A*β* protein-based active immunization is unique and shows significant protection against cognitive decline in the AD mouse model, which is accompanied by a reduction in the expression of key molecular biomarkers that are associated with oxidative stress and AD (Figure 4, Figure 5, Figure 6 and Figure 7). The importance of such active immunization in mice, and potentially in humans, is highlighted in comparison to other types of immunizations designed to treat AD in humans that showed limited clinical significance [30]. 

A reduction in A*β* deposits and plaque burden in the brain, especially in the hippocampal and cortical regions, is an important stage in preventing the development/progression of AD. Accordingly, the antigen-injected mice showed a significant average reduction in the levels of the most neurotoxic form of A*β* (i.e., A*β_42_* types) in blood-plasma (Figure 4C), whole brain (Figure 4D), and specific brain regions (Figure 4A,B). Furthermore, the current study demonstrated a degree of A*β* reduction that is compatible with the similarly observed reduction of A*β* in other AD mouse models that were treated with A*β*-targeted passive or active immunization [32]. The correlation between blood plasma/serum levels of A*β* and dementia are not always reliable in predicting the stage of AD in humans [33]. However, in AD-mouse models (like APP/PS1), a reduction of blood-plasma level of A*β_42_* serves as a good biomarker for evaluating the efficacy of an applied condition that aims at preventing/inhibiting the progression of AD-related brain pathologies [34]. Accordingly, compared to the control mice, the antigen-injected mice showed lower peripheral blood-plasma levels of A*β_42_* (Figure 4C) that correlated with the levels observed in whole brain and specific brain regions (Figure 4A,C,D). Thioflavin-S staining of the brain slices (Figure 4E,F) corroborated the data acquired for the A*β* levels (Figure 4A–D), although this staining is somewhat less specific than the antibody against A*β* in detecting A*β* plaque burden. Nevertheless, compared to the controls, the lower plaque burden demonstrated in the antigen-injected mice following the Thioflavin-S staining reproduced our previous same analysis performed on another transgenic AD mouse model [B6C3 Tg(APPswe,PSEN1dE9)85Dbo/J] that was injected with a similar MetO-rich antigen [20]. Specifically, in that study the antigen-injected mice showed a 28% lower plaque burden compared to controls [20], which was similar to the data obtained in the current study (Figure 4E,F). 

Astrocytes are important for homeostasis regulation of extracellular factors of the central nervous system that ultimately affect neuronal function and integrity. Through these cells’ function, the antioxidant defense of the surrounding neuronal cells is enhanced. However, under elevated oxidative stress conditions, the astrocytes may be damaged and fail to deliver their purpose. For example, lack of MsrA in *MsrA* knockout mouse caused structural damages to astrocytes, presumably due to the accumulation of MetO-proteins [26]. Apparently, the APP/PS1 mice exhibit noticeable levels of MetO-proteins in astrocytes in HPC, RSC, and EC brain regions, which were significantly diminished upon antigen immunization (Figure 5). This phenomenon may be attributed to the ability of the MetO-rich protein immunization to reduce oxidative stress that in turn lowers the cellular level of MetO. The signaling pathways and mechanisms that participate in this process are yet to be determined. Enhanced level of activated microglia may be indicative of the need to remove damaged neurons or accumulated forms of toxic A*β* in brain through the macrophagic function of microglia. The APP/PS1 mice exhibit elevated levels of activated microglia with an advanced age [35]. Thus, the ability of the current vaccination to significantly reduce these activated microglia levels (Figure 6) correlates well with the reduced A*β* levels and plaque burden observed in the antigen-immunized mice (Figure 4). 

Activation of the transcription factor Nrf2 causes its translocation to the nucleus, resulting in an enhanced transcription of antioxidant genes [22]. This function of Nrf2 is of particular interest for recent research aiming to determine how an increase of cellular antioxidant capability may protect against the development of AD. Our data show that Nrf2 is predominantly located in the nucleus of neuronal cells of antigen-immunized mouse brains compared with the controls (Figure 7). These data suggest that the brain cells of the antigen-immunized mice maintain a stronger antioxidant defense compared to the control: a phenomenon that supports the observation showing a superior protection against methionine oxidation in astrocytes of the antigen-injected mice (Figure 5). The biological events that are involved in the MetO-rich protein mediated activation of Nrf2 are yet to be determined. More research in this field is required to elucidate the details of the interaction between an exposure of cells to MetO-rich protein, active immunization, and Nrf2 activation. 

Recently, elastin-derived peptides (EDPs) have been shown to foster the overproduction of A*β* in a mouse model of AD [36,37,38]. Accordingly, it will be interesting to evaluate the possible beneficial effect of the current MetO-active immunization on reducing the A*β* levels in these mice. 

Further studies are expected to be completed in the future, aiming at evaluating the ability of the active MetO-rich protein immunization to protect the brain against aging-related abnormalities. To achieve this goal, the immunization will be performed in wild-type mice in a similar fashion that was described for the APP/PS1 mice. Thereafter, the mice will be evaluated for their memory and learning capabilities, using the depicted bio-behavioral tests. Subsequently, postmortem analyses will be conducted to assess the ability of this immunization to reduce the expression of molecular markers that are associated with the aging process. 

## 5. Conclusions

The current study demonstrates that active immunization with MetO-rich protein in AD-model mice mitigates their AD-associated phenotypes compared to the controls. It is suggested that the clearance of MetO-containing proteins (including MetO-A*β*) from blood-plasma and extracellular regions in the brain inhibits the progression of AD-related pathologies. The identity of the potential other toxic MetO-containing proteins that are cleared through this vaccination is yet to be determined. Nevertheless, the strong effect resulting by this treatment in alleviating cognitive decline and increased levels of AD markers in brain provides the foundation for pursuing this type of vaccination against AD in humans.

## 6. Patent

J.M., A.S.S. and F.P.G., have a patent application related to this work.

## Figures and Tables

**Table 1:** Treatments and analyses of APP/PS1 mice as function of their age.

Age (Weeks)	16	18	20	40	42
**Treatment**	1st Injection	2nd Injection	3rd Injection	Bio-behavioral testing	Euthanasia and post-mortem analyses on blood and brain tissues

Transgenic APP/PS1 mice were injected per each injection time with the adjuvant only (for the control mice; Alum, *n* = 9 mice), and adjuvant + antigen (for the experimental mice; Alum vol/vol with MetO-rich protein (50μg); *n* = 9 mice). At 40 weeks of age the mice were tested for their bio-behavioral performance (Morris Water Maze and Y-maze). At 42 weeks of age the mice were perfused, and euthanized, and postmortem analyses were performed (i.e., determining the levels of: A*β*, amyloid-plaque burden, protein-MetO, and activated microglia; determining Nrf2 cellular localization).

## Data Availability

Data is contained within the article and Supplementary Materials.

## Supplementary Materials

The following supporting information can be downloaded at: https://www.mdpi.com/article/10.3390/antiox11040775/s1, Figure S1: Western blot analysis showing detection of the antigen using the newly created rabbit anti-MetO antibody as the primary antibody (1:2000 dilution incubated for 1 h; kDa, Molecular mass markers shown in kilodaltons). Video S1: A representative control mouse performance in MWM (Figure 3E), Video S2: A representative Antigen-injected mouse performance in MWM (Figure 3E).

## References

[1] Selkoe D.J., Hardy J. (2016). The amyloid hypothesis of Alzheimer’s disease at 25 years. EMBO Mol. Med..

[2] Sultana R., Butterfield D.A. (2010). Role of oxidative stress in the progression of Alzheimer’s disease. J. Alzheimer’s Dis..

[3] Halliwell B. (2001). Role of free radicals in the neurodegenerative diseases: Therapeutic implications for antioxidant treatment. Drugs Aging.

[4] Terman A., Brunk U.T. (2006). Oxidative stress, accumulation of biological ‘garbage’, and aging. Antioxid. Redox Signal..

[5] Nunomura A., Perry G., Pappolla M.A., Wade R., Hirai K., Chiba S., Smith M.A. (1999). RNA oxidation is a prominent feature of vulnerable neurons in Alzheimer’s disease. J. Neurosci..

[6] Selkoe D.J. (2001). Alzheimer’s disease: Genes, proteins, and therapy. Physiol. Rev..

[7] Hardy J.A., Higgins G.A. (1992). Alzheimer’s disease: The amyloid cascade hypothesis. Science.

[8] Roychaudhuri R., Yang M., Hoshi M.M., Taplow D.B. (2009). Amyloid beta-protein assembly and Alzheimer disease. J. Biol. Chem..

[9] Nunomura A., Perry G., Aliev G., Hirai K., Takeda A., Balraj E.K., Jones P.K., Ghanbari H., Wataya T., Shimohama S. (2001). Oxidative damage is the earliest event in Alzheimer disease. J. Neuropathol. Exp. Neurol..

[10] Hirai K., Aliev G., Nunomura A., Fujioka H., Russell R.L., Atwood C.S., Johnson A.B., Kress Y., Vinters H.V., Tabaton M. (2001). Mitochondrial abnormalities in Alzheimer’s disease. J. Neurosci..

[11] Praticò D. (2008). Evidence of oxidative stress in Alzheimer’s disease brain and antioxidant therapy: Lights and shadows. Ann. N. Y. Acad. Sci..

[12] Dumont M., Lin M.T., Beal M.F. (2010). Mitochondria and antioxidant targeted therapeutic strategies for Alzheimer’s disease. J. Alzheimer’s Dis..

[13] Bitan G., Tarus B., Vollers S.S., Lashuel H.A., Condron M.M., Straub J.E., Teplow D.B. (2003). A molecular switch in amyloid assembly: Met35 and amyloid beta-protein oligomerization. J. Am. Chem. Soc..

[14] Näslund J., Schierhorn A., Hellman U., Lannfelt L., Roses A.D., Tjernberg L.O., Silberring L., Gandy S.E., Winblad B., Greengard P. (1994). Relative abundance of Alzheimer A beta amyloid peptide variants in Alzheimer disease and normal aging. Proc. Natl. Acad. Sci. USA.

[15] Kuo Y.M., Kokjohn T.A., Beach T.G., Sue L.I., Brune D., Lopez J.C., Kalback W.M., Abramowski D., Sturchler-Pierrat C., Staufenbiel M. (2001). Comparative analysis of amyloid-beta chemical structure and amyloid plaque morphology of transgenic mouse and Alzheimer’s disease brains. J. Biol. Chem..

[16] Dong J., Atwood C.S., Anderson V.E., Siedlak S.L., Smith M.A., Perry G., Carey P.R. (2003). Metal binding and oxidation of amyloid-beta within isolated senile plaque cores: Raman microscopic evidence. Biochemistry.

[17] Boutte A.M., Woltjer R.L., Zimmerman L.J., Stamer S.L., Montine K.S., Manno M.V., Cimino P.J., Liebler D.C., Montine T.J. (2006). Selectively increased oxidative modifications mapped to detergent-insoluble forms of A*β* and *β*-III tubulin in Alzheimer’s disease. FASEB J..

[18] Butterfield D.A., Boyd-Kimball D. (2005). The critical role of methionine 35 in Alzheimer’s amyloid beta-peptide (1–42)-induced oxidative stress and neurotoxicity. Biochim. Biophys. Acta.

[19] Triguero L., Singh R., Prabhakar R. (2008). Comparative molecular dynamics studies of wild-type and oxidized forms of full-length Alzheimer amyloid beta-peptides A*β*(1–40) and A*β*(1–42). J. Phys. Chem. B.

[20] Moskovitz J., Maiti P., Lopes D.H., Oien D.B., Attar A., Liu T., Mittal S., Hayes J., Bitan G. (2011). Induction of methionine-sulfoxide reductases protects neurons from amyloid β-protein insults in vitro and in vivo. Biochemistry.

[21] Stadtman E.R., Moskovitz J., Berlett B.S., Levine R.L. (2002). Cyclic oxidation and reduction of protein methionine residues is an important antioxidant mechanism. Mol. Cell. Biochem..

[22] Schmidlin C.J., Dodson M.B., Madhavan L., Zhang D.D. (2019). Redox regulation by NRF2 in aging and disease. Free Radic. Biol. Med..

[23] Oien D.B., Canello T., Gabizon R., Gasset M., Lundquist B.L., Burns J.M., Moskovitz J. (2009). Detection of oxidized methionine in selected proteins, cellular extracts and blood serums by novel anti-methionine sulfoxide antibodies. Arch. Biochem. Biophys..

[24] Hu J., Qin H., Gao F.P., Cross T.A. (2011). A systematic assessment of mature MBP in membrane protein production: Overexpression, membrane targeting and purification. Protein Expr. Purif..

[25] Webster S.J., Bachstetter A.D., Nelson P.T., Schmitt F.A., Van Eldik L.J. (2014). Using mice to model Alzheimer’s dementia: An overview of the clinical disease and the preclinical behavioral changes in 10 mouse models. Front. Genet..

[26] Pal R., Oien D.B., Ersen F.Y., Moskovitz J. (2007). Elevated levels of brain-pathologies associated with neurodegenerative diseases in the methionine sulfoxide reductase A knockout mouse. Exp. Brain Res..

[27] Gallagher J.J., Minogue A.M., Lynch M.A. (2013). Impaired performance of female APP/PS1 mice in the Morris water maze is coupled with increased A*β* accumulation and microglial activation. Neurodegener. Dis..

[28] Deng Y., Marsh B.M., Moskovitz J. (2019). Increased Levels of Protein-methionine Sulfoxide in Plasma Correlate with a Shift from a Mild Cognitive Impairment to an Alzheimer’s Disease Stage. Innov. Clin. Neurosci..

[29] Serneels L., Van Biervliet J., Craessaerts K., Dejaegere T., Horré K., Van Houtvin T., Esselmann H., Paul S., Schäfer M.K., Berezovska O. (2009). Gamma-Secretase heterogeneity in the Aph1 subunit: Relevance for Alzheimer’s disease. Science.

[30] Avgerinos K.I., Ferrucci L., Kapogiannis D. (2019). Safety and efficacy of active and passive immunotherapy in mild-to-moderate Alzheimer’s disease: A systematic review and network meta-analysis. Clin. Investig. Med..

[31] Iwasaki A. (2017). Immune regulation of antibody access to neuronal tissues. Trends Mol. Med..

[32] Wisniewski T., Goñi F. (2014). Immunotherapy for Alzheimer’s Disease. Biochem. Pharmacol..

[33] Janelidze S., Stomrud E., Palmqvist S., Zetterberg H., Van Westen D., Jeromin A., Song L., Hanlon D., Tan Hehir C.A., Baker D. (2016). Plasma β-amyloid in Alzheimer’s disease and vascular disease. Sci. Rep..

[34] Harach T., Marungruang N., Duthilleul N., Cheatham V., Mc Coy K.D., Frisoni G., Neher J.J., Fåk F., Jucker M., Lasser T. (2017). Reduction of A*β* amyloid pathology in APPPS1 transgenic mice in the absence of gut microbiota. Sci. Rep..

[35] Navarro V., Sanchez-Mejias E., Jimenez S., Muñoz-Castro C., Sanchez-Varo R., C Davila J., Vizuete M., Gutierrez A., Vitorica J. (2018). Microglia in Alzheimer’s Disease: Activated, Dysfunctional or Degenerative. Front. Aging Neurosci..

[36] Ma C., Su J., Sun Y., Feng Y., Shen N., Li B., Liang Y., Yang X., Wu H., Zhang H. (2019). Significant upregulation of Alzheimer’s β-amyloid levels in a living system induced by extracellular elastin polypeptides. Angew. Chem. Int. Ed..

[37] Ma J., Ma C., Li J., Sun Y., Ye F., Liu K., Zhang H. (2020). Extracellular matrix proteins involved in Alzheimer’s disease. Chemistry.

[38] Szychowski K.A., Skóra B., Wójtowicz A.K. (2021). Elastin-Derived Peptides in the Central Nervous System: Friend or Foe. Cell. Mol. Neurobiol..

