# Supplementary material for: Protective Effects against the Development of Alzheimer’s Disease in an Animal Model through Active Immunization with Methionine-Sulfoxide Rich Protein Antigen
Source: Antioxidants (Basel). 2022 Apr 13;11(4):775. doi: 10.3390/antiox11040775 (PMC9029927; doi:10.3390/antiox11040775)
Supplement: Supplementary file 1 [file antioxidants-11-00775-s001.zip › Supplementary Figure S1.pdf]

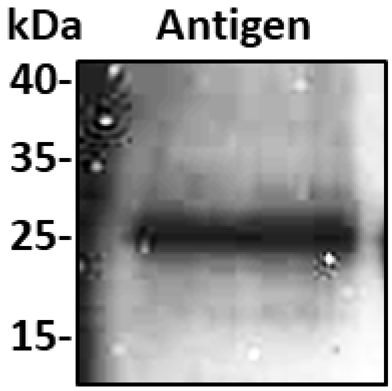

Figure S1: Western blot analysis showing detection of the antigen using the newly created rabbit anti-MetO antibody as the primary antibody (1:2000 dilution incubated for 1 h; kDa, Molecular mass markers shown in kilodaltons).
